# The Venom Repertoire of *Conus gloriamaris* (Chemnitz, 1777), the Glory of the Sea

**DOI:** 10.3390/md15050145

**Published:** 2017-05-20

**Authors:** Samuel D. Robinson, Qing Li, Aiping Lu, Pradip K. Bandyopadhyay, Mark Yandell, Baldomero M. Olivera, Helena Safavi-Hemami

**Affiliations:** 1 Department of Biology, University of Utah, Salt Lake City, UT 84112, USA; pradip.bandyopadhyay@gmail.com (P.K.B.); olivera@biology.utah.edu (B.M.O.); helena.safavi@utah.edu (H.S.-H.); 2 Eccles Institute of Human Genetics, University of Utah, Salt Lake City, UT 84112, USA; liqing850104@gmail.com (Q.L.); myandell@genetics.utah.edu (M.Y.); 3 School of Life Sciences and Technology, Institute of Protein Research, Tongji University, Shanghai 200092, China; aplv@tongji.edu.cn; 4 USTAR Center for Genetic Discovery, University of Utah, Salt Lake City, UT 84112, USA

**Keywords:** venom, conotoxin, *Conus*, cone snail, *Conus gloriamaris*

## Abstract

The marine cone snail *Conus gloriamaris* is an iconic species. For over two centuries, its shell was one of the most prized and valuable natural history objects in the world. Today, cone snails have attracted attention for their remarkable venom components. Many conotoxins are proving valuable as research tools, drug leads, and drugs. In this article, we present the venom gland transcriptome of *C. gloriamaris*, revealing this species’ conotoxin repertoire. More than 100 conotoxin sequences were identified, representing a valuable resource for future drug discovery efforts.

## 1. Introduction

**Figure 1:**
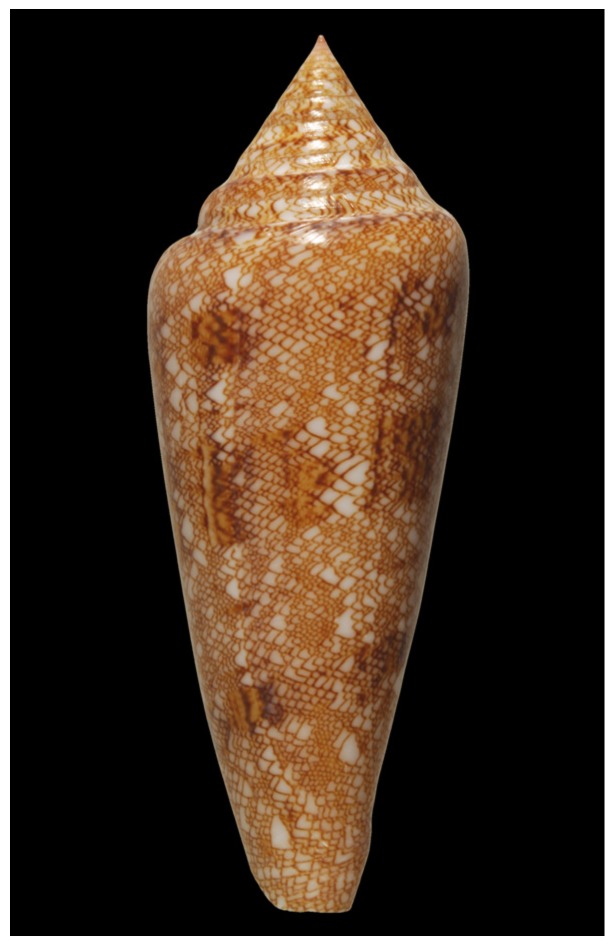
The shell of *Conus gloriamaris*. At one time, this was among the most prized natural history objects in the world.

For many years, the shell of the Glory of the Sea cone snail, *Conus gloriamaris*, was one of the most prized and valuable natural history objects in the world [1,2]. The shell is indeed remarkably beautiful (Figure 1), but it is perhaps its rarity that contributed most to its fame—for over two centuries, *C. gloriamaris* was known from only half a dozen specimens.

In 1837, Hugh Cuming, a British shell collector, found two live specimens on a reef on the island of Bohol, the Philippines [2]. Soon after, however, it would be reported that this reef, the only known location at which *C. gloriamaris* had been found alive, had been destroyed by an earthquake. Rumours followed that this already rare species had now been driven to extinction, and produced the obvious effect of making the few known specimens even more desirable. As recently as 1957, the number of known specimens was still only at two dozen. However, this would change as collectors entered into the waters of New Guinea and in 1969, a pair of SCUBA divers discovered over a hundred live specimens at Guadalcanal in the Solomon Islands [3].

Although still not considered to be abundant, *C. gloriamaris* is in fact reasonably widespread across the Indo-Pacific. It has been found between 5 and 300 m, but is rarely encountered in shallow water (above 100 m) [4], explaining its historical rarity. Nevertheless, it remains probably the single most famous seashell, and because of its beauty and historic significance, still attracts high prices from collectors.

Today, cone snails are attracting attention for an additional reason, the remarkable biomedical potential of their venom components. Each one of the ~750 species of the genus *Conus* is venomous (number of extant species according to WoRMS: http://www.marinespecies.org/). They use their venom for prey capture and defense [5]. Some species prey on fish, whilst others prey on marine worms or molluscs. *C. gloriamaris* is believed to be a mollusc-hunter [4]. Their venoms are complex mixtures typically containing more than 100 bioactive peptides (known as conotoxins). Furthermore, each species of *Conus* is thought to harbour an almost unique repertoire of conotoxins. Many conotoxins have unmatched potency and selectivity profiles at their respective molecular targets [6]. Additionally, while only a minute fraction of the estimated total number of conotoxins has been investigated to date, several have already proven valuable as research tools, drug leads, and drugs. ω-MVIIA, a conotoxin from the venom of *Conus magus*, is used for the treatment of chronic pain [7], while several others are under development for the treatment of various pathologies [8], including epilepsy, neuropathic pain, and diabetes.

Conotoxins are produced, by the cone snail, in a specialized venom gland. Conotoxin transcripts are ribosomally-translated into precursor peptides that undergo folding and post-translational processing before being secreted into the lumen of the venom gland [9]. In general, conotoxin precursor peptides are characterized by an *N*-terminal signal peptide followed by a propeptide region, and, encoded at the *C*-terminus, a single copy of the bioactive mature toxin.

Recent advances in high throughput ‘next-generation’ sequencing have made it possible to sequence the entire transcriptome of a tissue in a rapid and cost-effective manner. The application of this technology to the venom glands of *Conus* offers an avenue to acquiring a comprehensive picture of a species’ conotoxin repertoire [10,11,12,13].

The venom of *C. gloriamaris* has yet to be comprehensively studied. Only 10 conotoxin sequences have so far been reported from this species [14] and only two conotoxins (Gm9a and GmVIA) have been functionally characterised [15,16]. In this article, we present the venom gland transcriptome of *C. gloriamaris*, revealing the venom repertoire of this iconic species.

## 2. Results

Sequencing generated from *C. gloriamaris* whole venom gland RNA yielded a total of 42,602,912 demultiplexed raw reads (40,363,512 following adapter-trimming and quality-trimming/filtering). Using Trinity [17], 16,353 transcripts were assembled with an *E*_90_ of 238 (the number of transcripts that are supported by 90% of the expression data). Transcripts annotated as conotoxin precursors made up ~70% of the total expression data, as well as the bulk of the most highly expressed transcripts, i.e., all of the top 60 annotated transcripts were conotoxin precursors.

**Figure 2:**
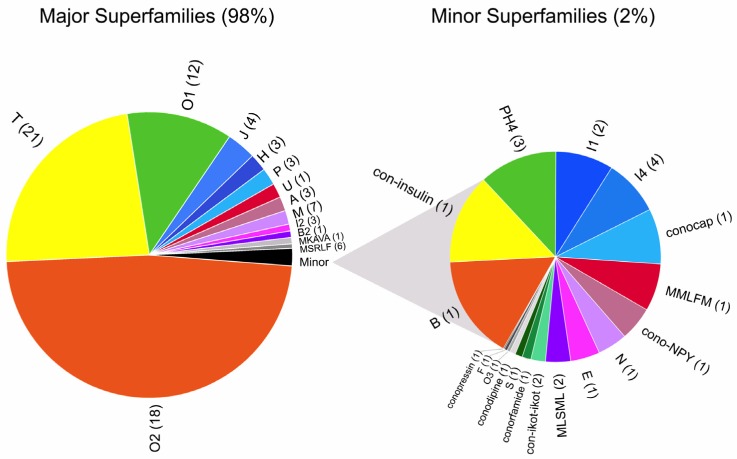
Distribution of toxin gene families in *C. gloriamaris*. Conotoxin expression is dominated by only a few conotoxin gene families. Values represent transcripts per million transcripts (TPM) counts obtained for all sequences belonging to the gene family. The number of individual sequences per toxin gene family is provided in parenthesis.

Conotoxin precursors can be grouped into gene families (or superfamilies), based on their signal peptide sequence identity [18]. A total of 31 distinct conotoxin gene families were identified in *C. gloriamaris*. Not all toxin gene families were expressed at equal levels. In fact, 95% of conotoxin expression was derived from just nine gene families (O2, T, O1, J, H, P, U, A, and M; from highest to lowest) (Figure 2).

From the 31 toxin gene families, a total of 108 individual conotoxin transcripts were identified. Again, there are substantial differences in the number of individual toxins per gene family. Almost all of the toxin gene families identified were represented by three or fewer individual toxins. Only five toxin gene families were represented by more than four individual conotoxin transcripts (O2, T, O1, M, and MSRLF) (Figure 2).

### 2.1. O2-Superfamily

**Figure 3:**
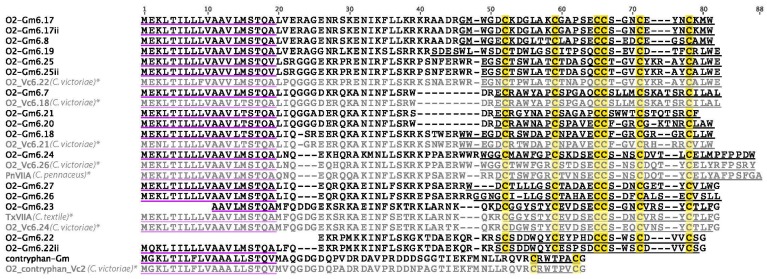
Sequence alignment of *C. gloriamaris* O2-superfamily precursor sequences. Precursor sequences of O2_Vc6.22, O2_Vc6.18, O2_Vc6.21, O2_Vc6.26, O2_Vc6.24, O2_contryphan_Vc2 [13], PnVIIA [20], and TxVIIA [19] are shown for comparison in grey and marked with *; Cys, yellow; Signal peptides are underlined in purple and predicted mature peptides are underlined in black/grey. This color scheme is used in all subsequent figures.

In *C. gloriamaris*, the O2-superfamily is the single most-highly expressed toxin gene family, accounting for close to half of the total conotoxin expression. It is also a diverse superfamily: 17 individual O2-superfamily conotoxins were detected (Figure 3). Of these, 16 have the typical type VI/VII cysteine framework (C-C-CC-C-C) and one belongs to a subclass known as contryphans. Of the two O2-superfamily conotoxins characterized so far (TxVIIA and PnVIIA), both appeared to be mollusc-specific in activity, producing strong paralytic effects in molluscs, but with no paralytic effects on arthropods or vertebrates [19,20]. Notably, for a number of the *C. gloriamaris* O2-superfamily conotoxins identified here, an identical or nearly identical sequence was previously described in *Conus victoriae* [13]. *C. victoriae* is a closely-related species belonging to the same subgenus (*Cylinder*), endemic to the coast of North-Western Australia. As is discussed below, a striking similarity to *C. victoriae* is observed for almost all of the toxin families identified.

### 2.2. T-Superfamily

**Figure 4:**
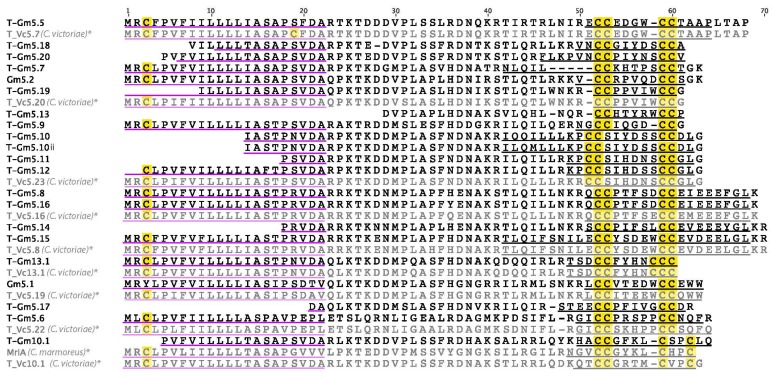
*C. gloriamaris* T-superfamily precursor sequences. *, precursor sequences of T_Vc5.7, T_Vc5.20, T_Vc5.23, T_Vc5.16, T_Vc5.8, T_Vc13.1, T_Vc5.19, T_Vc5.22, T_Vc10.1 [13], and MrIA [26] are shown for comparison.

In *C. gloriamaris*, the T-superfamily is the second most highly expressed toxin gene family, making up 22% of the total conotoxin expression. It is also the most diverse: 21 individual sequences were identified (Figure 4), including the previously reported Gm5.1 and Gm5.2 [21]. Nineteen of these had a type V cysteine framework (CC-CC), one a type XIII framework, and one a type I/X cysteine framework (CC-C-C). Several molecular targets, including presynaptic calcium channels [22], TTX-sensitive sodium channels [23], and somatostatin receptors [24], have been reported for conotoxins of the former subclass, while the toxins of the latter subclass are norepinephrine transporter inhibitors [25]. For several of the *C. gloriamaris* T-superfamily conotoxins identified here, an identical or nearly identical sequence was previously described in *C. victoriae*.

### 2.3. O1-Superfamily

**Figure 5:**
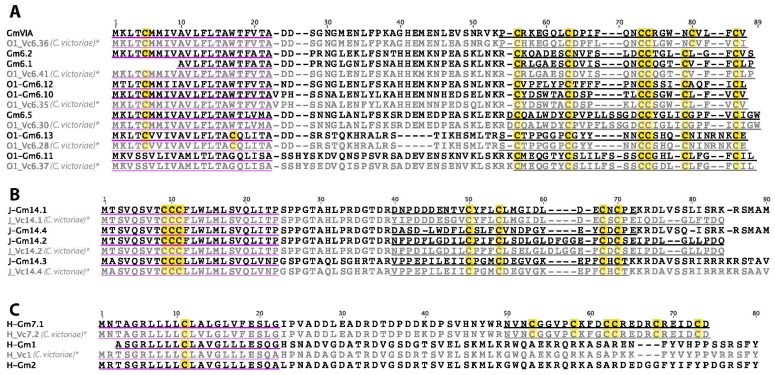
(**A**) *C. gloriamaris* O1-superfamily; (**B**) J-superfamily; (**C**) H-superfamily precursor sequences. *, precursor sequences of O1_Vc6.36, O1_Vc6.41, O1_Vc6.35, O1_Vc6.30, O1_Vc6.28, O1_Vc6.37, J_Vc14.1, J_Vc14.2, J_Vc14.4, H_Vc7.2, and H_Vc1 [13] are shown for comparison.

A total of 12 O1-superfamily conotoxin precursors were identified in *C. gloriamaris* (Figure 5A), including four sequences previously reported from this species (Gm6.1, Gm6.2, Gm6.5 [27], and GmVIA [28]). GmVIA is a δ-conotoxin, which delays the inactivation of voltage-gated sodium channels and produced convulsions in molluscs [28]. All O1-superfamily precursors from *C. gloriamaris* encode mature peptides with the typical type VI/VII cysteine framework, and all, except one low-expressed transcript, fit into the same δ/μ functional subclass as GmVIA. Again, for several of the *C. gloriamaris* O1-superfamily conotoxins identified here, an identical or close match was previously described in *C. victoriae*.

### 2.4. J-Superfamily

Four J-superfamily conotoxins were identified in the venom gland transcriptome of *C. gloriamaris* (Figure 5B). They are similar to the sequence previously identified in *C. victoriae* [13], but differ from the previously characterised J-superfamily conotoxin pl14a, which produces excitatory symptoms (shaking, barrel-rolling, and seizures) in mice on intracranial injection and was shown to be an inhibitor of the voltage-gated potassium channel subtype K_v_1.6 [29].

### 2.5. H-Superfamily

Both cysteine-rich (one), and cysteine-poor (two) H-superfamily conotoxins were identified (Figure 5C). The cysteine-poor conotoxins are both closely related to H-Vc1, previously identified in *C. victoriae*, while the predicted mature peptide of the cysteine-rich H-Gm7.1, differs only by a single residue from that of Vc7.2 from *C. victoriae*. The biological function is yet to be reported for any H-superfamily conotoxin.

### 2.6. P-Superfamily

**Figure 6:**
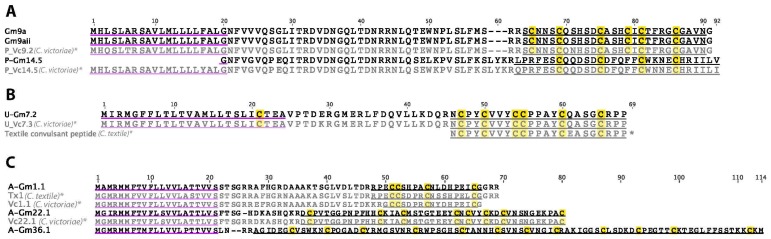
(**A**) *C. gloriamaris* P-superfamily; (**B**) U-superfamily; (**C**) A-superfamily precursor sequences. *, precursor sequences of P_Vc9.2, P_Vc14.5, U_Vc7.3, Vc22.1 [13], Tx1 [30], Vc1.1 [31], and the textile convulsant mature peptide [32], are shown for comparison.

Three P-superfamily transcripts were identified in the venom gland transcriptome of *C. gloriamaris* (Figure 6A). One of these is Gm9a (previously identified from *C. gloriamaris* [15]) and another appears to be an allelic variant of this sequence, differing by only a single synonymous substitution. Gm9a elicited hyperactivity and spasticity in mice on intracranial injection. The third precursor, P-Gm14.1, encodes a 30 residue mature peptide cysteine framework XIV (C-C-C-C). It is closely related to Vc14.5 from *C. victoriae*, differing in its predicted mature peptide by only three residues.

### 2.7. U-Superfamily

A single precursor sequence belonging to the U-superfamily was identified in *C. gloriamaris* (Figure 6B). The predicted mature peptide is identical to the previously described Vc7.3 from *C. victoriae* and differs by one residue from the “Textile convulsant peptide” from *C. textile*, which produces convulsions when injected intracranially in mice [32].

### 2.8. A-Superfamily

Three A-superfamily conotoxin precursors were identified in the *C. gloriamaris* venom gland transcriptome (Figure 6C). The most highly expressed A-superfamily sequence in *C. gloriamaris* was Gm1.1. The predicted mature peptide is similar to several previously described α-conotoxins and is likely to also be an inhibitor of neuronal subtypes of nicotinic acetylcholine receptors. Gm22.1 belongs to an unusual subclass that was previously described in *C. victoriae* [13]. A comparison of Gm22.1 with Vc22.1 is shown in Figure 6C. While there are minor differences in the signal and propeptide, the predicted mature peptides are identical between species. The third precursor shares the A-superfamily signal sequence, but appears to encode an unusually large (87 residues) mature peptide with 12 cysteines, and is unrelated to any previously described sequence.

### 2.9. M-Superfamily

**Figure 7:**
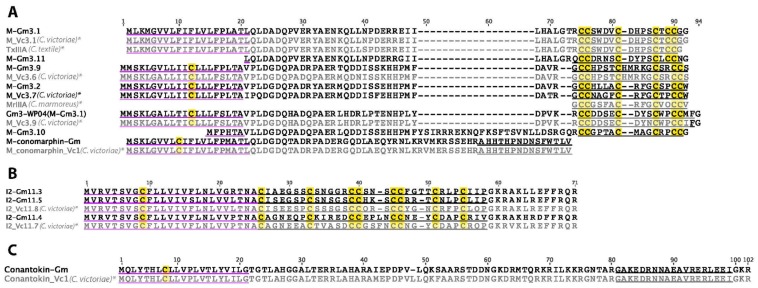
(**A**) *C. gloriamaris* M-superfamily; (**B**) I2-superfamily; (**C**) B-superfamily precursor sequences. *, precursor sequences of M_Vc3.1, M_Vc3.6, M_Vc3.9, M_conomarphin_Vc1, I2_Vc11.8, I2_Vc11.7, conantokin_Vc1 [13], TxIIIA [34], and the mature peptide of MrIIIA [35] are shown for comparison.

In *C. gloriamaris*, seven M-superfamily conotoxins were detected (Figure 7A), including the previously reported Gm3-WP04 (GenBank: JF510902.1). All, bar one, belong to the “mini-M” subclass [33], which produce excitatory symptoms when injected intracranially in mice, but for which a general molecular target is yet to be defined. The remaining sequence encodes a cysteine-free conomarphin.

### 2.10. I2-Superfamily

Three I2-superfamily conotoxins were identified in *C. gloriamaris* (Figure 7B). There are similarities to previously identified sequences, in particular those of *C. victoriae*, but there is little similarity to any of the I2-superfamily toxins with a known function (as potassium channel blockers [36,37,38]).

### 2.11. B2-Superfamily

**Figure 8:**
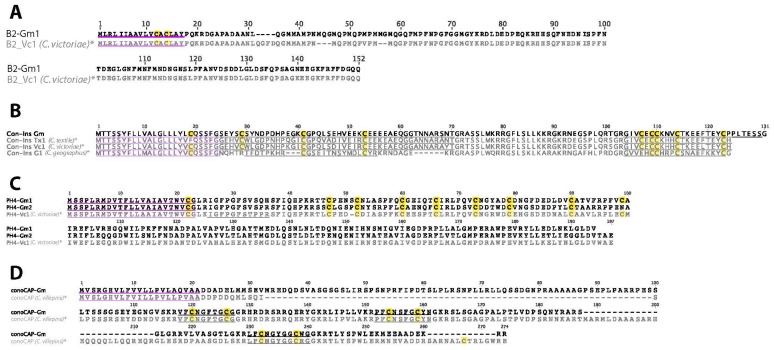
(**A**) *C. gloriamaris* B2-superfamil; (**B**) Con-insulin; (**C**) prohormone-4; (**D**) conoCAP precursor sequences. *, precursor sequences of B2_Vc1 [13], Con-Ins Tx1 [39], Con-Ins Vc1, PH4-Vc1 [40], and conoCAP [41] are shown for comparison.

A single B2-supefamily sequence was detected in *C. gloriamaris* (Figure 8A). The B2-“superfamily” refers to an unusual class of sequence that is found at a high frequency in the venom glands of all species of *Conus* examined [13]. The functional role of this class of sequences remains unclear.

### 2.12. B-Superfamily (Conantokins)

One conantokin was identified in the *C. gloriamaris* venom gland transcriptome (Figure 7C). Conantokins are cysteine-free peptides, some of which are antagonists of vertebrate *N*-methyl-D-aspartate receptor (NMDA) receptors [42]. Conantokin-Gm1 is very similar to the single conantokin sequence identified in *C. victoriae*. Minor differences are seen in the signal and propeptide, while the encoded mature peptides are identical between the two species.

### 2.13. Con-Insulin

A single venom insulin transcript was identified in *C. gloriamaris* (Figure 8B). Specialised insulins are a venom component of many species of *Conus* [39]. One of these, Con-Ins G1 from *C. geographus*, has been characterised, and binds to the vertebrate insulin receptor, inducing “insulin shock” in the fish prey.

**Figure 9:**
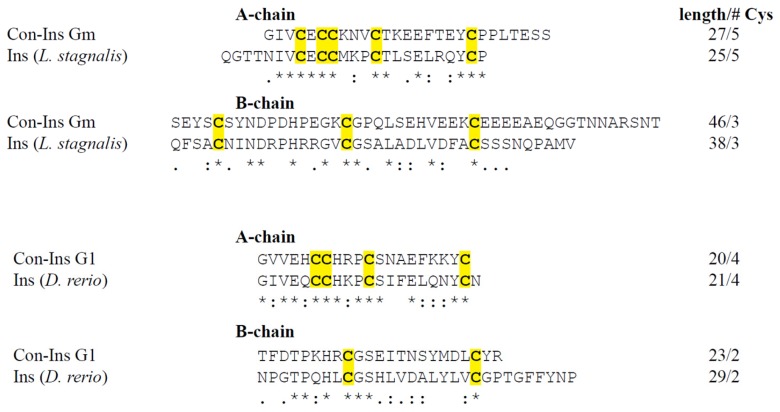
Similarity in cysteine arrangement, chain lengths, and amino acids between the A and B chains of Con-Ins Gm and an endogenous molluscan insulin, and Con-Ins G1 and an endogenous fish insulin. Comparative alignments of Con-Ins Gm and the giant pond snail *Lymnaea stagnalis* insulin 1 [Uniprot: P07223], and Con-Ins G1 from the venom of *C. geographus* and the zebrafish *Danio rerio* insulin [Uniprot: O73727]. Chain lengths and the cysteine number are shown following the sequence. Cysteines are highlighted in yellow. Post-translational modifications are omitted.

The venom insulin from *C. gloriamaris* belongs to the same gene family as Con-Ins G1 [43]. The predicted signal sequences of both venom insulins are highly similar, a characteristic of secreted venom components that belong to the same gene family. However, the predicted mature insulins differ significantly. The insulin from *C. gloriamaris* is longer and has an additional interchain disulfide bond. These differences can readily be rationalized by comparing the two venom insulins with the endogenous insulins of the zebrafish and the snail *Lymnaea stagnalis* (Figure 9). The *C. gloriamaris* insulin has the same organization and a greater sequence similarity to the *Lymnaea* insulin, while Con-Ins G1, the insulin from *C. geographus*, shows a much greater similarity to the zebrafish hormone. *C. geographus* is a fish-hunting species, while *C. gloriamaris* is a snail hunter. Thus, in each case, the structure of the venom insulin appears to reflect a strong selection for efficacy in the distinct prey of each species.

### 2.14. Prohormone-4

Two prohormone-4 precursors were identified in *C. gloriamaris* (Figure 8C). Prohormone-4 is a neuropeptide that was initially identified in the brain of the honeybee, *Apis melifera*, but was more recently identified as a venom component in some species of *Conus* [40].

A second class of prohormone-4-like precursors that do not appear to encode any mature peptide have been reported in all *Conus* examined to-date [40]. A transcript belonging to this class was also identified in *C. gloriamaris*.

### 2.15. I1-Superfamily

**Figure 10:**
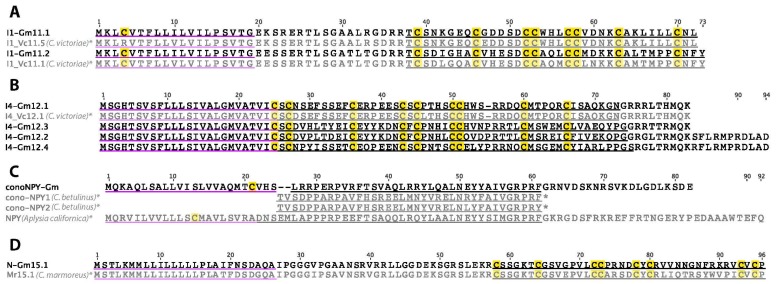
(**A**) *C. gloriamaris* I1-superfamily; (**B**) I4-superfamily; (**C**) cono-NPY; (**D**) N-superfamily precursor sequences. *, precursor sequences of I1_Vc11.5, I1_Vc11.1, I4_Vc12.1 [13], cono-NPY [45], and Mr15.1 [10] are shown for comparison.

Two I1-superfamily conotoxins were identified in *C. gloriamaris* (Figure 10A). Closely-related sequences were previously reported in *C. victoriae* [13]. The predicted mature peptide of Gm11.2 differs by a single amino acid to that of I1-Vc11.5. The I1-superfamily conotoxins characterized so far produce excitatory symptoms in mice, and one, RXIA, produces these through the agonism of voltage-gated sodium channels [44].

### 2.16. I4-Superfamily

Four I4-superfamily precursors were identified in *C. gloriamaris* (Figure 10B). The predicted mature peptide of I4-Gm12.1 differs from that of the previously described I4_Vc12.1 [13], by only two residues. No function has yet been ascribed to any conotoxin belonging to the I4-superfamily.

### 2.17. ConoCAP

A conoCAP precursor was identified in the *C. gloriamaris* venom gland transcriptome (Figure 8D). ConoCAPs are short single disulphide-containing peptides identified in the venom of some worm-hunting cone snails, which reduce the heart rate and blood pressure (in vertebrates) [41]. In contrast to other *Conus* venom peptides, multiple conoCAPs are encoded on a single long neuropeptide-like precursor. The *C. gloriamaris* conoCAP precursor encodes three individual mature peptides.

### 2.18. Cono-NPY

Cono-NPY was the name given to two peptides previously isolated from the venom of the worm-hunter *Conus betulinus* because of their similarity to neuropeptide-Y [45]. Here, we report, in the venom gland transcriptome of *C. gloriamaris*, a precursor that encodes a mature peptide with a sequence similarity to the previously reported cono-NPY peptides (Figure 10C). High expression in the venom gland and a secretory signal peptide are consistent with the role of this peptide as a toxin in the venom of *C. gloriamaris*. Furthermore, preliminary investigations indicate that other mollusc-hunting cone snails share closely related precursors in their venoms (unpublished observation).

### 2.19. N-Superfamily

A single N-superfamily precursor, Gm15.1, was found in the venom gland transcriptome of *C. gloriamaris* (Figure 10D). It shows a high sequence similarity to Mr15.1, one of the sequences previously identified in *C. marmoreus* [10]. The biological function is yet to be reported for any conotoxin of the N-superfamily.

### 2.20. E-Superfamily

**Figure 11:**
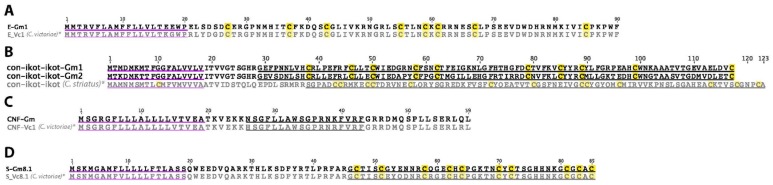
(**A**) *C. gloriamaris* E-superfamily; (**B**) con-ikot-ikot; (**C**) conorfamide; (**D**) S-superfamily precursor sequences. *, precursor sequences of E_Vc1, S_Vc8.1 [13], con-ikot-ikot [46], and CNF_Vc1 [47] are shown for comparison.

A single E-superfamily sequence was identified in *C. gloriamaris* (Figure 11A). The alignment of this sequence with that of *C. victoriae* reveals seven differences across the 90 residue precursor. No biological function has been reported for any conotoxin of this “superfamily”.

### 2.21. Con-ikot-ikot

Two con-ikot-ikot precursor sequences were identified in *C. gloriamaris* (Figure 11B). Con-ikot-ikots are a class of conotoxin predominantly found in the venoms of fish-hunters. Con-ikot-ikot from *Conus striatus* inhibits AMPA receptor channel desensitization [46] and was recently used as a tool to co-crystalize and reveal the activation mechanism of this receptor [48]. However, the *C. gloriamaris* sequences differ substantially, such that a similar function would not be expected.

### 2.22. Conorfamide

A single conorfamide precursor sequence was identified in *C. gloriamaris* (Figure 11C). It bares a striking similarity to CNF-Vc1, the conorfamide identified in *C. victoriae* [47], with no differences in the signal peptide, one in the *C*-terminal propeptide, and two in the mature peptide. CNF-Vc1 caused excitatory symptoms in mice on intracranial injection, as well as the depolarization of sensory neurons.

### 2.23. S-Superfamily

A single S-superfamily precursor sequence was identified in the venom gland transcriptome of *C. gloriamaris* (Figure 11D). Two conotoxins from the S-superfamily have been characterized so far; one is an inhibitor of serotonin receptors [49], while the other is a nicotinic acetylcholine receptor inhibitor [50].

### 2.24. Conodipine

**Figure 12:**
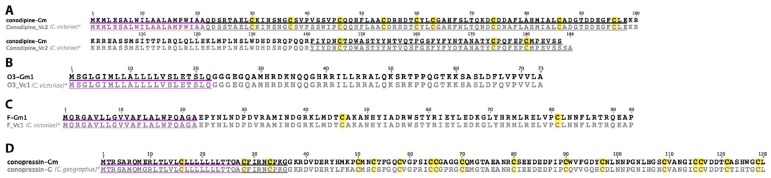
(**A**) *C. gloriamaris* conodipine; (**B**) O3-superfamily; (**C**) F-superfamily; (**D**) conopressin precursor sequences. *, precursor sequences of conodipine, O3_Vc1, F_Vc1 [13], and conopressin-G [11] are shown for comparison.

The precursor sequence of a single conodipine was detected in the *C. gloriamaris* venom gland transcriptome (Figure 12A). Conodipines are a class of secretory phospholipase-A_2_ enzyme found in *Conus* venoms.

### 2.25. O3-Superfamily

A single precursor sequence belonging to the O3-superfamily was identified in *C. gloriamaris* (Figure 12B). In contrast to most previously reported O3-superfamily sequences, it lacks cysteines and appears to encode a cysteine-free mature peptide. A single cysteine-free O3-superfamily sequence has been previously identified in *C. victoriae*, to which the *C. gloriamaris* sequence is clearly closely related, differing by only three residues across the entire 73 residue precursor.

### 2.26. F-Superfamily

A single F-superfamily sequence was identified in *C. gloriamaris* (Figure 12C). Similar to the E-superfamily, this “superfamily” is defined by one precursor sequence, each from *C. marmoreus* [10] and *C. victoriae* [13], and no biological function has been reported. The alignment of the *C. gloriamaris* sequence with that of *C. victoriae* reveals complete identity across the entire peptide precursor.

### 2.27. Conopressin

A single conopressin transcript was identified (at low expression levels) in the venom gland transcriptome of *C. gloriamaris* (Figure 12D). Conopressins, analogues of the mammalian hormone vasopressin, have been described in the venoms of *Conus* [51]. Gonopressin-Gm is closely related to conopressin-G from *C. geographus*, and the predicted mature conopressin peptide is identical.

### 2.28. Putative Conotoxins (MKAVA, MSRLF, MMLFM, MLSML)

**Figure 13:**
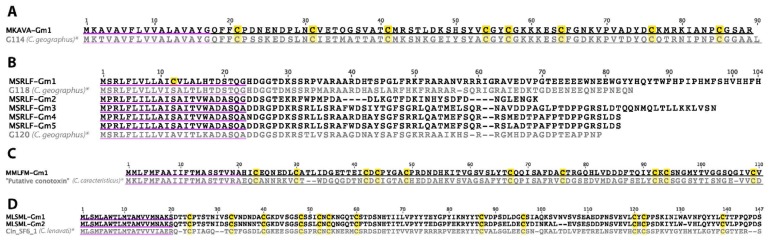
(**A**) *C. gloriamaris* MKAVA-superfamily; (**B**) MSRLF-superfamily; (**C**) MMLFM-superfamily; (**D**) MLSML-superfamily precursor sequences. *, precursor sequences of G114, G118, G120 [52], *C. caracteristicus* ‘putative conotoxin’ (GenBank: B0L0Y6.1), and Cln_SF6_1 [54] are shown for comparison.

Four additional putative conotoxin gene families were identified in the *C. gloriamaris* transcriptome: The class of transcript we temporarily refer to as the “MKAVA-superfamily” (Figure 13A) was previously reported in a *C. geographus* venom gland transcriptome [52], but was mis-annotated as the I1-superfamily. In *C. gloriamaris*, a single transcript sharing the same signal peptide and a closely related predicted mature peptide sequence, was identified at a very high expression level. A preliminary examination of other species has revealed that related transcripts are very widespread in *Conus* (unpublished observation).

Several transcripts sharing the same signal peptide as “new superfamily 1”, also previously reported in *C. geographus* [52], were identified in *C. gloriamaris* (Figure 13B), some at very high expression levels. To avoid ambiguity, we temporarily refer to this group of sequences as the “MSRLF-superfamily”. Similar to what was observed in *C. geographus*, the *C. gloramaris* sequences appear to encode cysteine-free mature peptides. A preliminary examination of other species has revealed that this superfamily is very widespread and diverse in *Conus* (unpublished observation).

The “MMLFM-superfamily” was initially represented by a single sequence from the worm-hunter *Conus caracteristicus* (GenBank: B0L0Y6.1), with other sequences recently reported in several worm-hunting species [53]. A single transcript, sharing the same general precursor structure, signal peptide sequence, and cysteine framework of this class was identified, at a reasonably high expression, in *C. gloriamaris* (Figure 13C), indicating that this class of transcripts is not limited to worm-hunters. A preliminary examination of other species has revealed that related transcripts are widespread in *Conus* (unpublished observation).

Two transcripts, which we will temporarily refer to as the “MLSML-superfamily”, were identified (Figure 13D). These shared a secretory signal peptide sequence and reasonably high expression levels. The predicted mature peptides are large and each contained 12 cysteine residues. These appear to be closely related to a transcript recently reported in the venom gland transcriptome of *Conus lenavati* (Cln_SF6_1) [54], and again, a preliminary examination of other species has revealed that related transcripts are widespread in *Conus* (unpublished observation).

## 3. Discussion

The original characterization of the crude venom of *C. gloriamaris* [28] revealed that this species’ venom is particularly enriched in a single component, δ-conotoxin GVIA. Consistent with this, GVIA is one of the most highly expressed individual toxins in our transcriptomic dataset. In fact, in all members of the subgenus *Cylinder* that have been examined so far, δ-conotoxins are highly expressed [13]. A comparison of a selection of the δ-conotoxins from *C. gloriamaris* to some from two other species in this subgenus, *C. textile* and *C. victoriae*, is shown in Table 1 δ-conotoxin GmVIA and other molluscan δ-conotoxins cause hyperactivity in the envenomated snail prey [28,55]; it has been suggested that the function of the extreme excitatory effects of this venom component is to prevent prey from escaping (since the envenomated snail is observed to flail in an uncontrolled, seizure-like manner). After this, the prey snail is often extended out of its shell, and therefore remains easily accessible to the predator cone snail (instead of the natural tendency of snails to withdraw deep into their shells at the first sign of danger).

**Figure 14:**
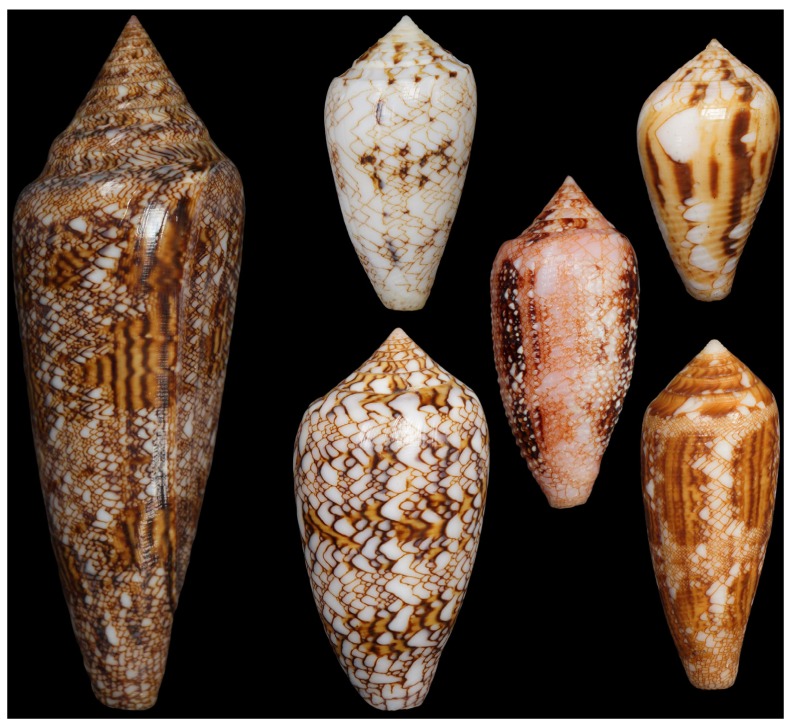
*C. gloriamaris* and some other species in the subgenus *Cylinder*. Left, *C. gloriamaris*; Second column, *C. victoriae* (Top), *C. textile* (Bottom); Third column, *Conus legatus*; Fourth column, *Conus retifer* (Top), *Conus aureus* (Bottom).

**Figure 15:**
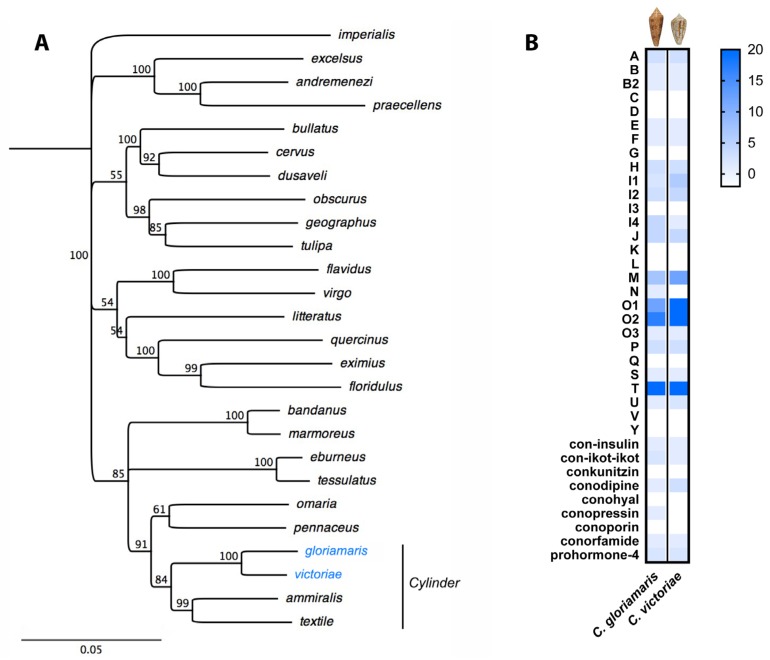
*Conus gloriamaris* shares a near-identical toxin gene family repertoire to the closely-related species *Conus victoriae*. (**A**) Phylogenetic tree of various *Conus* species highlighting close relatedness of *C. gloriamaris* and *C. victoriae* (subgenus *Cylinder*). Neighbor-joining tree of concatenated 12S, 16S and COI sequences was generated (Jukes-Cantor model) using Geneious version 8.1.3. Sequences were obtained from GenBank. *Conus imperialis* was used as an outgroup. Branch labels show consensus support (%); (**B**) A comparison of the expressed toxin gene family repertoire of *C. gloriamaris* and *C. victoriae*. Heat map is indicative of individual conotoxin number in each gene family.

In all three species, a venom insulin is also expressed (Table 1). The presence of venom insulins suggests that after the initial uncontrolled excitatory motor response, the snail would become hypoglycemic and therefore transformed into a more quiescent state (while remaining outside its shell). Other components of the venom, including the α-toxins that block nicotinic acetylcholine receptors, would then presumably cause a generalized flaccid paralysis. Thus, the results suggest at least two phases of prey capture: a state of hyper-excitability that guarantees that the envenomated prey is unable to withdraw into its shell, and a transition to a hypoglycemic state and flaccid paralysis. When the prey is quiescent and paralyzed outside its shell, *C. gloriamaris* can begin to engulf and digest the now helpless, easily accessible prey. Together, our data suggest a shared prey-capture strategy for *C. gloriamaris* and other species in *Cylinder* for capturing their snail prey, which involves the use, among other toxins, of both excitatory δ-conotoxins and venom insulins. It seems likely that other intriguing insights into the chemical strategies used by these snails may emerge as these venoms are studied further.The venoms of mollusc-hunting species of *Conus* have so far not been heavily studied. The venom gland transcriptomes of three other species, *C. marmoreus* [10], *C. victoriae* [13], and *C. episcopatus* [56] have been reported. Of these, *C. victoriae* is of the same subgenus, *Cylinder*, as *C. gloriamaris* (Figure 14 and Figure 15A). As indicated above, these species appear to share a similar venom repertoire. Indeed, clear similarities emerge on a comparison of the toxin repertoire of *C. gloriamaris* to that of *C. victoriae* [13]. Of the 23 conotoxin gene families detected in *C. victoriae* [13,40,47], all are present in *C. gloriamaris*. Only two additional conotoxin gene families were detected here in *C. gloriamaris* (N and conopressin) that were looked, for but not found, in *C. victoriae*. Both were present here as single transcripts at relatively low expression levels (626.1 and 22.5 TPM, respectively). The increased sensitivity provided by larger raw read numbers (40,363,512 reads (Illumina) in this study versus 701,536 reads (454) in the prior *C. victoriae* study) may have facilitated their detection here. Both species also shared the absence of certain conotoxin gene families (C, D, G, I3, K, L, V, and Y). Thus, the two species share what appears to be a near identical toxin gene family repertoire.

The two species also share a similar total number of conotoxin transcripts. A total of 97 conotoxin transcripts (from the 23 conotoxin superfamilies reported in both species) were identified here in *C. gloriamaris*, compared with 119 reported in *C. victoriae* [13,40,47]. It should be noted that the venom glands of multiple individuals of *C. victoriae* were used, while in this study, the venom gland of a single individual of *C. gloriamaris* was used. Thus, the population level genetic polymorphism, as reported in other *Conus* species [57], potentially contributes to the slightly greater total number of transcripts reported for *C. victoriae*.

There are also clear similarities in the distribution of transcripts between toxin gene families (Figure 15B). Those, in *C. gloriamaris*, with a high diversity of transcripts (T, O2, O1, and M) also exhibit a high diversity in *C. victoriae* [13]. Likewise, those of a low diversity, where only one or two transcripts exist, are shared between the two species. A comparison of the absolute number of individual conotoxins in each gene family is given in Figure 15B (comparisons based on the expression level were not appropriate since the *C. victoriae* data were generated from a cDNA library that was normalized).

Thus, on multiple levels, it is clear that the venom repertoire of *C. gloriamaris* is remarkably similar to that of the closely related *C. victoriae*. However, does this similarity extend to the individual toxin level? Of the 97 *C. gloriamaris* conotoxin transcripts (from the 23 conotoxin gene families reported in both species), 14 encode mature peptides with identical matches in *C. victoriae*, and numerous others encode mature peptides with near-identical matches (i.e., one to a few residues difference). To our knowledge, this is the highest rate of identical toxins between any species of *Conus* so far examined, and it indicates that certain species *can* share substantial overlap in venom composition. While the similarities are striking, it is important to not lose sight of the fact that more than 85% of the venom components of *C. gloriamaris* do *not* have an identical match in *C. victoriae*. The relatively subtle differences between the venom repertoires of the two closely related species are largely seen at the mature peptide level and may be a reflection of the differences in prey specialization. For example, *C. gloriamaris* is a deep-water offshore species, while *C. victoriae* occupies shallower marine habitats, and while both are mollusk-hunters, differences in their specific prey might be expected.

The venoms of mollusc-hunting species of the cone snail have proven their potential as a source of therapeutically-relevant peptides [8] (e.g., χ-MrIA and Vc1.1). Moreover, to the drug discoverer, the comparison between the venoms of *C. gloriamaris* and *C. victoriae* highlights two important points: i) That the venom of each species of *Conus* represents a near-unique library of natural products; and ii) the venom repertoire of certain species can mirror that of closely related species, essentially representing a library of naturally-occurring analogues. This type of knowledge can be valuable in guiding targeted drug discovery efforts from *Conus* venoms.

The toxin repertoire of *C. gloriamaris* (summarized in Table 2) should offer rich grounds for the discovery of new functions. Of the 108 conotoxins identified, a molecular target can be confidently predicted for only a handful. Even for these, differences in the primary structure may impact subtype selectivity. For example, A-Gm1.1, which shows a sequence similarity to known nAChR blockers, would be predicted to have a similar function, but perhaps a novel subtype selectivity profile. Others belong to classes which are demonstrably bioactive (i.e., Gm9a, contryphan-Gm, CNF-Gm, U-Gm7.2, Gm10.1, and each the conotoxins of the M-superfamily), but for which a specific molecular target or mechanism of action is yet to be defined. However, the vast majority of the conotoxins presented still remain completely uncharacterized. Given the successful history of the small fraction of conotoxins characterized so far, it seems probable that a further exploration of this *C. gloriamaris* conotoxin library has the potential to yield new research tools, if not drug leads or therapeutics.

## 4. Materials and Methods

A single live adult specimen of *C. gloriamaris* was collected from Balicasag Island in the Philippines using gill nets—a fine mesh net was laid out on the sea bottom, at a depth of ~120 m, for approximately three months, after which time the net was raised and the colonized molluscs were collected.

The venom gland was dissected (~7 cm in length) and stored in RNA-later, before being transferred for storage at −80 °C. Total RNA extraction was performed using the Direct-zol RNA extraction kit (Zymo Research, Irvine, CA, USA), with on-column DNase treatment, according to the manufacturer’s instructions. cDNA library preparation and sequencing was performed by the University of Utah High Throughput Genomics Core Facility: Total RNA quality and quantity were first validated on an Agilent 2200 TapeStation (Agilent Technologies, Santa Clara, CA, USA). A dual-indexed library was constructed with the Illumina TruSeq Stranded mRNA Sample Prep Kit with oligo (dT) selection and an average insert size of approximately 150 bp. The library was validated on an Agilent 2200 TapeStation and using a qPCR assay (Kapa Biosystems Library Quantification Kit for Illumina, Boston, MA, USA), and was pooled in a batch of 13 samples. 125 cycle paired-end sequencing was performed on an Illumina HiSeq2000 instrument (San Diego, CA, USA) at an 80% standard cluster density.

Adapter trimming of de-multiplexed raw reads was performed using fqtrim [71], followed by quality trimming and filtering using prinseq-lite [72]. Error correction was performed using the BBnorm ecc tool, part of the BBtools package. Trimmed and error-corrected reads were assembled using Trinity (version 2.2.1) [17] with a k-mer length of 31 and a minimum k-mer coverage of 10. Assembled transcripts were annotated using a blastx [73] search (E-value setting of 1e-3) against a combined database derived from UniProt (downloaded April 2015), Conoserver [14], and an in-house conotoxin library. Transcripts per million transcripts (TPM) counts were generated using the Trinity RSEM [74] plugin (align_and_estimate_abundance) and expression data were analysed using the trinity utilities: abundance_estimates_to_matrix and contig_ExN50_statistic. An in-house script was used to extract conotoxin transcripts, trim to open-reading frame, and discard redundant and partial sequences. The final list of assembled conotoxin transcripts was then manually examined using the Map-to-Reference tool of Geneious, version 8.1.7 [75].

Conotoxin precursor sequences from this Transcriptome Shotgun Assembly project have been deposited at DDBJ/EMBL/GenBank [accession: GFNK00000000]. The version described in this paper is the first version, GFNK01000000. Raw sequencing data has been deposited in the NCBI sequence read archive [SRA accession: SRR5499408].

## Figures and Tables

**Table 1:** δ-conotoxins and venom insulins of *C. gloriamaris* and other species of the subgenus *Cylinder.*

**δ-Conotoxins**		**Primary Structure**
GmVIA		VKPCRKEGQLCDPIFQNCCRGWNCVLFCV
Vc6.36 (*C. victoriae*) [13]		GKPCHKEGQLCDPFLQNCCLGWNCVFVCI
Tx6.1 (*C. textile*)		QVKPCRKEHQLCDLIFQNCCRGWYCVVLSCT
Gm6.1		CRLGAESCDVISQNCCQGTCVFFCLP
Vc6.41 (*C. victoriae*) [13]		CRLGAESCDVISQNCCQGTCVFFCLP
TxVIA (*C. textile*) [55]		WCKQSGEMCNLLDQNCCDGYCIVLVCT
TxVIB (*C. textile*) [19]		WCKQSGEMCNVLDQNCCDGYCIVFVCT
**Venom Insulins**		**Primary Structure**
Con-Ins Gm	A-chain	GIVCECCKNVCTKEEFTEYCPPLTESS *
	B-chain	SEYSCSYNDPDHPEGKCGPQLSEHVEEKCEEEEAEQGGTNNARSNT *
Con-Ins Vc1 (*C. victoriae*) [40]	A-chain	GIVCECCKHHCTKEELTEYCH
	B-chain	HVCWLGDPNHPKGICGPQLSDIVEIRCEEKEAEQGGANNARAYT *
Con-Ins Tx1 (*C. textile*) [39]	A-chain	GIVCECCKHHCTKEEFTEYCH
	B-chain	GEHVCWLGDPNHPQGICGPQVADIVEIRCEEKEAEQGGANNARANT *

Predicted or known mature peptides are shown. *, *C*-terminal amidation (other post-translational modifications are not predicted).

**Table 2:** Functional diversity encoded in the venom gland transcriptome of *Conus* *gloriamaris***.** Each conotoxin superfamily is divided into groups according to the cysteine framework, with the number identified in *C.* *gloriamaris* and a summary of biological activity associated with each group indicated.

Superfamily	Cysteine Framework	# Identified in *C. gloriamaris*	Associated Activities	Reference
A	I	1	nAChRs inhibitors, GABA_B_ receptor agonists, α1-adrenoceptor inhibitor	[25,58,59]
-	XXII	1	N.D.	-
-	XXXVI	1	N.D.	-
conantokin (B)	Cysteine-free	1	NMDA receptor inhibitors	[42]
Con-insulin	-	1	Insulin receptor agonists	[39]
conodipine	-	1	Phospholipase-A_2_	[60]
cono-NPY	Cysteine-free	1		[45]
conorfamide	Cysteine-free	1	Convulsions in mice (IC), sensory neuron depolarization	[47]
conopressin	Single disulfide	1	Vasopressin receptor agonists	[51]
I1	XI	2	Na_V_ agonists	[44]
I2	XI	3	K^+^ channel modulators	[37,38]
J	XIV	4	nAChR inhibitor and K_V_ inhibitor	[29]
M	III	7	Excitatory symptoms in mice (IC)	[35,61,62]
conomarphin (M)	Cysteine-free	1	N.D.	-
(M)	Single disulfide	1	N.D.	-
O1	VI/VII	12	Na_V_ agonists, K_V_ blockers, Na_V_ blockers or Ca_V_ blockers	[63,64,65,66]
O2	VI/VII	17	Neuronal pacemaker modulators	[19]
contryphan (O2)	Single disulfide	1	Ca^2+^ channel modulators	[67,68,69]
O3	Cysteine-free	1	N.D.	-
P	IX	2	Hyperactivity and spasticity in mice (IC)	[15]
-	XIV	1	N.D.	-
S	VIII	1	5-HT_3_ receptor inhibitor, nAChR inhibitor	[49,50]
T	V	19	Na_V_ inhibitor, presynaptic Ca^2+^ channel inhibitor (or GPCR modulator), sst3 GPCR antagonist	[22,23,24]
-	XIII	1	N.D.	-
-	X	1	Noradrenaline transporter inhibitors	[25]
U	VI/VII	1	Convulsions in mice (IC)	[70]

Ca_V_, voltage-gated calcium channel; GABA, γ-aminobutyric acid; GPCR, G protein-coupled receptor; IC, intracranial injection; K_V_, voltage-gated potassium channel; nAChR, nicotinic acetylcholine receptor; Na_V_, voltage-gated sodium channel; N.D., not determined; NMDA, *N*-Methyl-D-aspartate; sst, somatostatin. Other gene families detected for which there is no reported associated activity (B2, E, F, H, I4, N, con-ikot-ikot, MKAVA, MSRLF, MMLFM, MLSML) are omitted from this table.
